# Perception and Acceptance of HPV Vaccination Among Women Treated for Cervical Intraepithelial Neoplasia: An Evidence-Based Narrative Review

**DOI:** 10.3390/jcm14248859

**Published:** 2025-12-15

**Authors:** Vasilios Lygizos, Rafaela Panagopoulou, Vasilios Pergialiotis, Eleni Sivylla Bikouvaraki, Sofoklis Stavros, Periklis Panagopoulos, Chrysi Christodoulaki

**Affiliations:** 1 First Department of Obstetrics and Gynecology, Alexandra Hospital, Medical School, National and Kapodistrian University of Athens, 11528 Athens, Greecepergialiotis@yahoo.com (V.P.); 2 Third Department of Obstetrics and Gynecology, University General Hospital “ATTIKON”, Medical School, National and Kapodistrian University of Athens, 12462 Athens, Greece; 3 Laboratory of Cell and Gene Therapy, Centre of Basic Research, Biomedical Research Foundation of the Academy of Athens (BRFAA), 11527 Athens, Greece; 4 Department of Obstetrics and Gynecology, Chania General Hospital “St. George”, 73300 Chania, Greece

**Keywords:** human papillomavirus (HPV), cervical intraepithelial neoplasia (CIN), HPV vaccination, adjuvant immunization, vaccine acceptance, patient perception, secondary prevention, gynecologic oncology, behavioral determinants, physician recommendation

## Abstract

High-risk human papillomavirus (HPV), including types 16–18, is the established cause of cervical intraepithelial neoplasia (CIN) and invasive carcinoma of the cervix. While preventive vaccination is highly effective in preventing infection from becoming reconstituted following treatment of existing disease, its use among cervical intraepithelial neoplasia (CIN)-positive females has remained sporadic. The following review provides an update on the current state of evidence about the acceptance, awareness, or perception of HPV vaccination by women following a diagnosis or treatment of CIN. Methods: A narrative synthesis of literature from the publication period of 2010 to 2025 was performed on PubMed, Scopus, and Google Scholar. Surveys that quantified literature on post-CIN vaccination attitudes, risk perceptions, or behavioral factors were considered. Results: Acceptance levels varied from 20–95% across all continents. The highest acceptance levels (≥80%) among the populations belong to the European and Oceanian groups, followed by moderate acceptance among the North Americans (60–80%), which was influenced by financial costs, misconceptions, and sociocultural stigmas. Several systemic-level features in Europe and Oceania have been shown to be consistently associated across these regions with high acceptance rates. These features include public funding of HPV vaccine delivery universally in these regions and reminder and recall systems established in their electronic health records. In these two regions, provider recommendation demonstrates particular significance because there is follow-up care after treatment of CIN. In these regions, mass awareness about HPV conducted in conjunction with their cervical screening programs increases baseline knowledge and favorability towards HPV vaccination. The lowest levels (20–70%) of awareness of HPV diseases and vaccination programs among Asians and Africans can be attributed to obstacles that include misconceptions about fertility concerns. In the case of Asia, there are various socially ingrained stigma factors that contribute to the poor awareness and acceptance levels. These factors include the possibility of being perceived as promiscuous, embarrassment linked to STI conditions, as well as the possibility of rejection from partners and in-laws. In particular regions, there might be stigmas attached to HPV vaccination that cause tension within married women who perceive the vaccine as an indicator of being unfaithful. Also, distrust from the general community has been driven by past incidents, including the halting of proactive HPV vaccine recommendations in Japan in 2013. Moreover, there are numerous myths concerning infertility and menstruation linked to poor vaccine acceptance. The key determinant of acceptance levels was physician endorsement, lack of knowledge of the association of HPV-CIN, or the belief that there is no need for vaccination after treatment. Conclusion: The acceptance of HPV vaccination among women following CIN is influenced by educational level, the structure of the healthcare system, and sociocultural factors. Incorporating evidence-based cervical vaccination counseling into follow-up care after biopsy could help increase its acceptance and prevent recurrent high-grade lesions.

## 1. Introduction

Cervical intraepithelial neoplasia (CIN) can be considered the precursors of cervical cancer and are almost exclusively caused by infections of high-risk types of the human papillomavirus (HPV). The method of excisional therapy has been proven effective in eliminating high-grade lesions of the cervix entirely but has also been observed to confer women an increased lifelong risk of recurrence of CIN2+, which can be attributable to the persistence of latent infection of high-risk HPV. Various large observational studies and meta-analyses have been used to provide supportive information regarding the role of adjuvant HPV vaccine therapy in the secondary reduction in the risk of recurrence of CIN2+ of the cervix by at least 60–70%. However, the post-CIN treatment acceptability of the HPV vaccine remains suboptimally low across the global context [1,2].

### 1.1. Adjuvant HPV Vaccination After Surgical Treatment for CIN2/3

HPV vaccination has recently been proposed as an adjuvant prophylaxis strategy following surgical excision of high-grade cervical intraepithelial neoplasia (CIN2/3) [3]. Persistent or recurrent HPV infection remains the principal cause of post-treatment recurrence, with approximately 10–15% of women developing residual or new high-grade lesions despite adequate excision and negative surgical margins. Adjuvant vaccination enhances immune response, preventing reinfection by vaccine-covered HPV types and potentially reducing viral persistence in latent sites [4,5]. A growing body of evidence, including multiple systematic reviews and meta-analyses, demonstrates a 60–70% reduction in the risk of recurrent CIN2+ among women vaccinated perioperatively compared with unvaccinated controls. Sand et al.’s subsequent pooled analyses confirmed that vaccination—whether administered before or within one year after excision—significantly decreases the incidence of histologically confirmed recurrence, particularly for HPV-16/18–related lesions [2]. These findings have led major professional societies, including ESGO, ASCCP, and CDC, to recognize adjuvant HPV vaccination for immunization as a promising adjunct to standard post-excisional surveillance in eligible women up to age 45. This reflects a shift toward secondary prevention, transforming the management of CIN from a purely surgical intervention to an integrated immunopreventive approach that addresses both eradication of existing disease and protection against future infection [6].

### 1.2. Challenges in Real-World Uptake After CIN Treatment

Although the existing data on adjuvanted HPV vaccination has been highly encouraging in preventing recurrence of high-grade cervical lesions (CIN2+), it is astounding that scant few women actually receive this vaccination after treatment of CIN2/3. The lack of coverage achieved has been estimated variably, often <30–40% in regions with otherwise well-executed vaccination programs. The discrepancy between existing proof of concept and lack of practice does not indicate failure among efficacy trials, but lack of awareness, psychosocial engagement, or both. Many of these individuals consider vaccination following surgical removal of the lesion as redundant, as they often consider it an indication of complete cure of the disease. There also remain misconceptions among them concerning vaccine safety, fertility, or age indication.

From a healthcare perspective, there could be missed opportunities regarding vaccination counseling after treatment follow-up visits, which could be owing to the lack of a physician recommendation, an established predictor of acceptance. Moreover, barriers from the psychosocial–informational domain all contribute jointly to undermine the effectiveness of an established intervention by which a significant recurrence of cancer could be averted. Taking into account the concerns, emotional states, and educational levels of women would thus be pivotal in exploring the complete preventive benefit by establishing a relationship of post-CIN care involving HPV vaccination.

Owing to the existing discrepancy between the findings of clinical research and implementation practices, comprehension of the ‘human factor’ of how HPV vaccination can be readily received by society has become a matter of relevance in public health. The group of interest, that is, women diagnosed with cervical intraepithelial neoplasia (CIN), represents a specifically interested group of individuals exposed to heightened levels of psychological vulnerability, often facing anxiety, stigmatization, or the fear of disease recurrence. The findings of this research will be significant in understanding various attitudes of females concerning HPV vaccination after diagnosis of cervical intraepithelial neoplasia. What follows represents a description of the issue of interest, its relevance, which will help frame the research question of this research. The current review will focus on synthesizing the existing knowledge on factors of attitudes of females concerning HPV vaccination after diagnosis of cervical intraepithelial neoplasia.

Despite robust evidence that adjuvant HPV vaccination reduces CIN2+ recurrence by 60–70%, uptake remains low. Understanding post-treatment acceptance is therefore clinically essential.

## 2. Materials and Methods

The current review has been planned as an evidence-based narrative synthesis in accordance with SANRA quality criteria (Scale for the Assessment of Narrative Review Articles). It does not constitute a systematic review. The overall objective of the present narrative comprehensive review has been to identify and critically analyze all existing evidence from 2010 to 2025 on women’s views and acceptance of the HPV vaccine following treatment for CIN. The current narrative comprehensive review has used both quantitative and qualitative information to offer an overall perspective that considers clinical as well as system-level determinant factors.

The literature search was carried out on the search engines of Pubmed, Scopus, Embase, and Web of Science using the Boolean search terms: (“HPV vaccination” OR “human papillomavirus vaccine”) AND (“cervical intraepithelial neoplasia” OR “CIN” OR “cervical dysplasia”) AND (“acceptance” OR “attitude” OR “perception” OR “awareness” OR “knowledge” OR “determinant” OR “predictor”). The bibliographies of important guidelines and reviews from ESGO-EFC, WHO, and ACIP were also searched. For inclusion in the analysis, only peer-reviewed literature published in English and including human subjects was considered suitable. The complete search strategy appears in Supplementary Table S1.

We included studies published between 2010 and 2025 that focused specifically on women diagnosed or previously treated for cervical intraepithelial neoplasia (CIN) and that reported at least one of the following: HPV vaccination acceptance, awareness, attitudes, perceptions, behavioral factors, or determinants influencing vaccination after CIN treatment. Both quantitative and qualitative studies were eligible. Only peer-reviewed articles in English were included. On the other hand, we excluded studies that focused on the general population without CIN-specific data. These studies did not report outcomes related to post-CIN vaccination behavior, conference abstracts without full text, commentaries, editorials, animal studies, duplicated datasets, and papers not written in English.

Trials were included that showed original data on HPV vaccine acceptance, awareness, and perceptual patterns in women who had been previously treated for CIN or cervical precancer. Studies were included if they reported at least one of the following: acceptance rates, determinants, awareness, risk perception, or post-treatment vaccination behaviors. These trials also had to have quantifiable outcomes like immunization rates, acceptance levels, and determinants. Studies that included general population trials without direct relevance to immunization acceptance post-CIN treatment, animal trials, abstract data from conventions and symposiums, commentary sections, and repeated data were excluded. The search included publications from January 2010 to March 2025. Two independent reviewers independently examined the titles and abstract sections of all records for the trial inclusion criteria and obtained the full text of potentially relevant trials. In both trials, the data collected included authors’ details, year of publication, country of research, size of trial population, nature of research design, trial population data, acceptance levels, determinants of immunization acceptance, and context of the trial.

### Study Screening and Selection

All records identified through the database search were screened in two stages. First, two independent reviewers examined titles and abstracts to assess potential eligibility. Full texts were then obtained for all articles that met the initial criteria or where eligibility was uncertain. Discrepancies between reviewers were resolved through discussion, and when necessary, a third senior reviewer was consulted to reach consensus. The final set of included studies was determined after full-text evaluation.

A total of 498 records were identified across all databases after removal of duplicates. After title and abstract screening, 117 articles were retrieved for full-text evaluation. Ultimately, 56 studies met the eligibility criteria and were included in the narrative synthesis. Of these, 34 were original research articles, and 22 were review-type articles, including systematic reviews, narrative reviews, guideline papers, and meta-analyses that included CIN-specific post-treatment HPV vaccination data.

Given the heterogeneity of designs across the included studies, it was not possible to conduct a meta-analysis. However, an assessment of the quality of research methodology on an adapted set of critical appraisal principles for observational and qualitative research has been carried out. This approach aimed to substantiate the current evidence level and did not eliminate evidence of lower quality, as foreseen by the principles of narrative synthesis.

For a cohesive analysis framework, the data were plotted on two intersecting axes. The first axis represented geographic divisions by continents: Europe, North America, Asia, Latin America, and Africa. The approach enabled analysis of acceptance rates in the context of local variations in acceptance of vaccines as well as healthcare infrastructure. The second axis represented types of determinants based on a structure adapted from the socioecological model. The socioecological model included four levels: individual, interpersonal, system, and societal. At the individual level, factors included age, educational level, parity, prior knowledge of HPV infections, and beliefs about recurrent infections. The interpersonal factor included physician influence, partner/spouse influence, family and friend influence, or peer influence. The system factors were the cost of vaccines covered by healthcare services, vaccines accessible at healthcare centers, reminder services for HPV vaccinations, and easy access to subsequent care. At the societal level, there were factors of stigma against HPV-related infections in society due to ignorance and stigma. The structure enabled two-way analysis of both qualitative and quantitative data.

The findings were presented in tables and figures. The acceptance levels in the regions, the determinants of immunization, and the reduction in recurrence with adjuvant immunization post-CIN treatment were presented. The quantitative findings from larger trials such as the SPERANZA project and the Danish nationwide prospective cohort were synthesized with qualitative findings on awareness levels, attitudes, and implementation challenges. Inconsistencies were examined with respect to healthcare system types and disease acceptance.

All information used for writing the critique has been obtained from existing research literature. No new data has been collected. The article, therefore, did not require ethical consideration, as it involved neither human nor animal participants.

During manuscript preparation, generative AI tools were used exclusively for editing and reference organization. No AI tools were used for study selection, data extraction, analysis, interpretation, or the formulation of scientific conclusions.

## 3. Results

### 3.1. HPV Vaccination and Cervical Intraepithelial Neoplasia

Although HPV prophylactic vaccination was conceived as a tool of primary infection prevention, an increasing body of evidence shows its effectiveness in secondary infection prevention after cervical intraepithelial neoplasia (CIN) treatment. From a biological perspective, this strategy relies on the induction of immune memory, which effectively neutralizes HPV viral particles, thus preventing reinfection with oncogenic types covered by the vaccine. The vaccination could also increase local surveillance of the cervical transformation zone, thus preventing viral persistence.

It is known in fact that, after an effective cervical intraepithelial lesion removal, women still retain a high risk of acquiring cervical carcinoma throughout their entire life [1,2,4].

The effectiveness of adjuvant HPV vaccination after CIN2+ has been demonstrated by a number of randomized controlled trials and cohort studies. Meta-analyses of more than 20,000 CIN2/3-treated patients show a 60–70% reduced recurrence risk in the vaccinated group versus controls.

This estimate derives from previously published systematic reviews and meta-analyses, not from the present narrative review. The meta-analysis by Di Donato et al. (Vaccines 2021) [7] included 11 individual studies, while the Danish nationwide pooled analyses evaluated several large population-based cohorts. Together, these published analyses encompass more than 20,000 women treated for CIN2/3, which is the evidence base behind the commonly cited 60–70% reduction in recurrence.

Various clinical trials, including VIVIANE, FUTURE I/II, PATRICIA, and data collected from follow-up cohorts of vaccinated populations in Italy, Denmark, and Australia, prove that immunization pre- or post-excisions effectively lowers the occurrence of a follow-up incident of CIN2+. The vaccination efficacy has also been proven by follow-up analyses of more than a decade after immunization [1,2].

Across the analyzed literature, adjuvant HPV vaccination following excisional treatment for cervical intraepithelial neoplasia (CIN) was consistently associated with a marked reduction in disease recurrence. In the Italian SPERANZA prospective study, Ghelardi et al. observed a 60% decrease in CIN2+ recurrence among women vaccinated within twelve months after conization compared with unvaccinated controls (recurrence rates 1.7% vs. 4.3%) [8]. The Danish nationwide cohort of over 17,000 women reported by Sand et al. demonstrated a 45% lower risk of histologically confirmed CIN2+ recurrence following post-treatment vaccination, adjusted hazard ratio = 0.55 (95% CI 0.42–0.73) [3]. Similar findings were supported by the meta-analysis of Di Donato et al. (2021), which pooled more than 20,000 treated patients and confirmed a relative risk reduction of approximately 65% for recurrent high-grade lesions in vaccinated versus unvaccinated women [7]. Smaller prospective cohorts, including the German study by Jentschke et al. and the Korean trial by Kang et al., reinforced these outcomes, reporting recurrence reductions ranging between 50% and 70% [9,10]. Collectively, these results strengthen the hypothesis that vaccination after surgical treatment provides an effective secondary-prevention benefit beyond primary immunization, particularly against HPV-16/18-related lesions.

### 3.2. Current Guidelines’ Recommendations

Taking into consideration this evidence base, current guidelines from leading gynecologic oncologic societies, including the European Society of Gynecological Oncology (ESGO), the American Society for Colposcopy and Cervical Pathology (ASCCP), as well as the Centers for Disease Control and Prevention (CDC), recommend HPV vaccination in women post-CIN2/3 treatment up until age 45 years. These recommendations are consistent with the American Cancer Society guideline, which supports broad adult eligibility for HPV vaccination [11]. The ESGO consensus statement firmly advocates the role of adjuvant vaccination after cervical surgery on the premise of established efficacy and safety. The guidelines recommend co-administration of vaccination within the first year after cervical surgery [3].

### 3.3. Implementation and Patient Acceptance

Although there is strong clinical evidence supporting the use of adjuvant vaccination, its application on a broader level still requires improvement. The key implementing factor in this process is the acceptance of the preventive measure by the patients. Many females find surgical removal of lesions curative. Also, there is less awareness of the risks of recurrence. Clinicians’ efforts, along with proper information about the population, can thus become definitive steps in implementing this preventive measure.

### 3.4. Knowledge and Awareness Among Women with CIN

Even after decades of public education regarding this disease issue, there is still a lack of awareness of the etiological relationship of human papillomavirus (HPV) infection with cervical intraepithelial neoplasia (CIN). Many females with abnormal Pap tests or newly identified CIN do not understand that the HPV is the etiological factor leading to cervical dysplasias as well as the development of cervical carcinoma [4]. The lack of awareness can sometimes produce questions among individuals regarding the justification of vaccination against HPV following treatment. Common misconceptions continue among various groups. Some women feel that HPV vaccination is unnecessary after infection or the onset of CIN, as it is only beneficial as a preventive measure given to adolescents or unexposed females. Some others raise unjustified concerns that the vaccine could affect fertility, menstrual cycles, or induce an autoimmune disease. This is encouraged by misinterpretations on the internet. Some women consider HPV vaccination a treatment of the infection itself. This confuses realistic expectations from vaccination [4].

The level of awareness and knowledge is strongly related to education, screening participation, and socioeconomic status. Educated women with regular participation in the cervical screening program show higher levels of awareness for HPV transmission, vaccination efficacy, and recurrence rates. Lack of health literacy, financial constraints, and limited access to preventive services have been consistently found to be associated with poor awareness despite clear susceptibility to misconceptions. There remains an identified need for efforts focused on education to address gaps within these groups [4,5].

The clinician’s explanation, as well as the timing of communicating this information, plays an important role in building patients’ comprehension and decision-making. Evidence shows that personalized explanations provided during colposcopy or follow-up visits after biopsy can significantly increase vaccination acceptance, especially when framed by physicians not as an additional measure but as an inherent step in recovery [5]. Regional variation in awareness. Geographic and cultural variations also affect levels of awareness, as shown in Table 1. Results from Northern and Western Europe show a higher level of awareness of the role of HPV in cervical disease, while Southern and Eastern Europe demonstrate less awareness of HPV and more myths. The awareness of both HPV and its vaccination in Asian countries has been inconsistent, with good levels in urban and educated communities but a lack of awareness in rural areas owing to various stigmas, lack of accessibility, and distorted information channels in the healthcare settings [5,6,12].

### 3.5. Attitudes, Beliefs, and Emotional Factors

The diagnosis of cervical intraepithelial neoplasia (CIN) can cause significant emotional distress, including anxiety, guilt, or shame, mainly attributed to its association with an STI. Also, the diagnosis can be considered not only a medical issue but also a reflection of an individual’s behavior by many women. This can be attributed to the stigma of HPV infection, which can be worsened by an inadequate explanation of the association of HPV with cervical carcinoma by practitioners, thus making it difficult for individuals to feel comfortable with medical interventions like vaccination [6].

Such emotional responses are of key importance in the determination of risk perceptions as well as the willingness of an individual to take preventive measures. Female patients feeling fear or apprehension regarding the possibility of recurrence of diseases will or will not take the vaccination on account of denial, or could do the opposite, which is take the vaccination as a means of reclaiming their autonomy. Research shows that the concept of vaccination as an enabling act of claiming autonomy in protecting oneself from disease, and not as an indication of having been exposed, enhances emotional acceptance or openness [6,12].

Trust in healthcare professionals and institutions is found repeatedly as a key factor in accepting vaccination. For women, belief in their gynecologist, colposcopist, or the national health authorities is significant in accepting HPV vaccination [12]. Clear communication, care coordination, and endorsement by reputable organizations can help alleviate doubts created by myths. Perceived lack of clarity, rapid consultation visits, or lack of follow-up care contribute to skepticism among those carrying an emotional toll of diagnosis.

Various qualitative research among differing populations identifies some common themes encompassing the emotional experience of females post-CIN treatment as follows: “I wish I’d known earlier”, “Fear of recurrence”, “Concerns about side effects”, “Concerns about it potentially being too late for vaccination”.

The afore-mentioned quotes depict the true essence of regret, hope, and fear of patients. The concerns can easily be countered by directly dealing with them through counseling. Cultural practices, as well as the dynamics of the family, also influence vaccination choices. For example, in more collectivist cultures, acceptance of vaccination can be conditional on the approval or influence of spouses or the family, while in more individualist societies, autonomy or freedom of choice will be considered. The attitudes of one’s spouse concerning vaccination, attitudes of society concerning sexual health, or religious convictions can be the driving or inhibiting force. Recognizing the culture’s framework can help the practitioner adapt strategies in giving precepts on HPV vaccination by embedding it into the sociocultural environment of the vaccinated individual [6,12].

## 4. Discussion

The persistent infections by oncogenic HPV types such as HPV 16, 18, 31, 33, 45, 52, and 58 represent the prime underlying causal factor for cervical carcinogenesis. These types account for 90% of global cervical cancer cases altogether. HPV 16 and 18 alone account for almost 70% of the aggressive types. However, the low-risk types 6 and 11 were found to account for the majority of genital warts [7,8,9,10,21,22,23,24]. CIN stands for cervical intraepithelial neoplasia. These lesions represent a continuum of precursor lesions that include epithelial immaturity and varied nuclear atypias. Based on the 2012 Lower Anogenital Squamous Terminology (LAST), CIN 2 and CIN 3 lesions represent high-grade squamous lesions of the epithelia confirmed by positive immunostaining for p16. These lesions imply high-grade infections that transform and pose a high malignant potential [25].

Infection lasting above 12 months poses a substantial potential for aggressive CIN 3 lesions. Therefore, the biological imperative of prevention, both at the levels of infection and reinfection, stands amplified.

### 4.1. Pathogenesis and Prevention Integration

The continuum of HPV infection to Invasive Carcinoma offers an ideal rationale for an adjuvant approach to vaccines. Viral integration leads to disrupting the E2 regulatory elements with an attendant, uncontrolled expression of oncogenes E6/E7 that degrade proteins p53 and Retinoblastoma proteins that promote genomic instability [12]. The persistent viral epithelial lesions also maintain the low-grade lesions, and subsequent reinfection of the regeneration transformation zone in an excised lesion may initiate additional foci of dysplasia. This cycle prevents new infections and reinfections with OPV-types of HPV [24].

Thus, post-treatment immunization does not merely complement excisional therapy—it biologically closes the causal loop of HPV-driven carcinogenesis. This integration of molecular pathogenesis with preventive intervention epitomizes precision public health.

### 4.2. Factors That Influence Vaccination Acceptance and Adherence

Whether or not to vaccinate against HPV post-operatively for CIN treatment remains an intricate process involving various factors. Informed mainly by psychographic factors such as increased age and lower educational attainment as negative determinants of acceptance, given a better understanding of overall healthcare concepts and familiarity with gynecologists’ practice [26,27,28,29]. In contrast, reduced immunization levels have been found in the elderly population that may have difficulties gaining access to healthcare services due to geographical or socioeconomic circumstances. The underlying healthcare infrastructure of cost effectiveness and implementation within an existing schedule of care has been found to influence adherence [30,31].

Financial issues are also important. The presence of government-supported immunization programs or those covered by insurance shows a much higher completion rate than those that require patients to pay out of pocket. Adding HPV immunization codes to existing surgical and follow-up billing protocols effectively shifts it from an elective preventive treatment to a new standard of care, as expected. The inclusion of reminder notices via automatic notifications in the patient’s electronic medical records or simply via text has already increased compliance by 20 to 40%, underscoring that it requires more than providing knowledge alone—it needs facilitation [14,30]. By adding immunization as the last step of recovery, as an optional preventive treatment, outcomes remain much better.

### 4.3. Behavioral Models and Predictors of Acceptance

Immunization choice following CIN treatment can be explained using the Health Belief Model and the Theory of Planned Behavior [15,32]. As mentioned in the Health Belief Model, personal perceptions of disease susceptibility and the benefits of immunization, as well as cues such as practitioner recommendations, serve as stimuli for preventive actions [29]. As applied to TPB theories, behavioral intention emerges only on the basis of attitudes toward immunization and internalized beliefs about an individual’s control over immunization [16,17,33,34,35,36]. Since these women comprehend that persistent HPV infections may recur due to latent viral reservoirs within the transformation zone of the cervix, the umbrella of cervical mucosa, their preventive strategy to immunization increases [18,19,37,38].

The persistent HPV infection leads to integration of the viral DNA into the host’s basal keratinocytes. This expression produces two proteins, E6 and E7. These proteins act by inactivating tumor suppressor proteins p53 and Rb, respectively [18]. These scientific facts highlight the important role of immunization post-therapy. The objective of immunization here is not the treatment of existing lesions but the prevention of reinfection of a biological area that has already been primed for malignant change. The tertiary preventive approach here is provided by the HPV vaccine.

**Figure 1:**
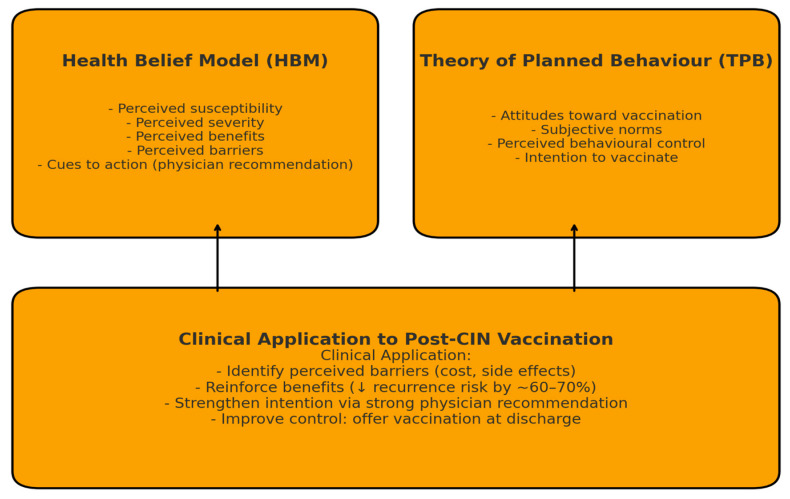
Behavioral models influencing HPV vaccine acceptance after CIN treatment. The arrow indicates the directional flow from individual-level perceptions (perceived susceptibility, perceived benefits, perceived barriers, and self-efficacy) toward the final behavioral intention to vaccinate, as described by the Health Belief Model and Theory of Planned Behavior.

The practical implementation of these frameworks of behavior makes it possible to create personalized strategies of counseling. For women who have high perceived barriers to vaccines, such as being expensive or having side effects, healthcare providers should highlight the high safety records of vaccines administered to hundreds of thousands of women worldwide [13,30,39,40,41,42,43]. For those with high perceived benefits but low self-efficacy, healthcare providers may need to offer logistical assistance, such as immunization, at discharge (Figure 1).

### 4.4. Digital Health and Dynamics of Misinformation

The digital information environment has a dramatic influence on attitudes toward healthcare. Social networking sites such as Facebook and online fora have proliferated misinformation on HPV vaccines, often perpetuating myths of infertility, autoimmune disease, or sexual disinhibition as adverse consequences of such immunization [31,44,45,46,47]. In contrast to the negative influence of online misinformation discussed above, online physician-led support groups and online healthcare-related websites increase trust levels in vaccines.

Findings of durable immunity following a single-dose regimen exist mostly through several landmark studies. The KEN SHE trial has proven the efficacy of single-dose HPV in protecting against new infections in Kenyan adolescents and young women. Studies in Costa Rica and India spearheaded by PATH also showed the strength of the immune response through the detection of antibodies up to 10 years post-immunization [30,31,47,48]. Evidence from the Rwandan national single-dose study also supports the sustained immunogenicity of the vaccine and its high effectiveness at the community level. Model-driven research studies, as presented by Brisson et al. in 2022 [49], showed the possibility of maintaining the protective effect of the vaccine beyond two decades through single-dose regimens. Evidence from the VIVIANE and Kjaer trials also supports the persistence of neutralizing antibody levels even at reduced dosages [14,50].

### 4.5. Physician Endorsement and Professional Responsibility

Among all factors influencing HPV vaccine acceptance, the endorsement of a physician’s role ranks supreme [34,44]. In gynaecologic oncology, the physician’s role as both a healthcare provider and an educator holds irreplaceable value. The physician’s strong indication of HPV immunization in the context of treatment completion significantly boosts acceptance. Lack of a strong indication triples the odds of refusal [33,44].

This emphasizes the importance of the professional ethics of providing a balanced perspective. Standardized immunization cues integrated into an electronic medical record system encourage uniformity. In addition, immunization discussions based on ESGO and ASCCP recommendations of immunization until age 45 and optimally within 12 months post-excisions establish immunization recommendations within evidence-derived guidelines [27,51,52]. These principles are also aligned with the ASCCP risk-based management framework, which integrates vaccination considerations into post-treatment follow-up strategies [53]. Continuing education for colposcopists and oncologists includes communication frameworks, shared decision-making, and cultural competence.

### 4.6. Health-System Integration and Cost-Effectiveness

The addition of HPV immunization to existing prevention strategies for cervical cancer can improve both efficiency and long-term effectiveness. Cost-effectiveness models have found that adding HPV testing to immunization during colposcopy visits or post-excisions has an incremental cost-effectiveness ratio of less than USD 15,000 per QALY in high-resource regions [21,54]. These approaches offer both recurrence prevention and new infection prevention simultaneously.

The WHO position paper of 2022 supporting single-dose immunization regimes marks the dawn of a new approach to implementation on a global scale [1,17,43]. The latest UpToDate analysis supports the notion that two- and single-dose immunization regimens retain immunogenicity at least as high as a three-dose regimen while offering substantial economic advantages by reducing implementation costs on a larger scale [48].

From a policy perspective, reimbursement mechanisms and bundled payments should favor integration. Public–private collaborations involving the relevant ministries of health, manufacturing companies, and professional organizations may provide for subsidized value chains, especially for tertiary hospitals in resource-poor settings [30,31].

### 4.7. Barriers and Facilitators

Despite substantial evidence supporting adjuvant HPV vaccination, real-world uptake remains suboptimal. Common barriers include limited awareness, fear of side effects, distrust toward the pharmaceutical industries, and misconceptions that surgery alone guarantees a cure [16,33]. Structural obstacles—fragmented care pathways, limited vaccine availability, and lack of provider recommendation—further compound the issue [18,19].

Conversely, facilitators are clear: strong physician endorsement, culturally sensitive counseling, and logistical convenience. Studies demonstrate that offering on-site vaccination in colposcopy or oncology clinics doubles completion rates compared to external referrals [16,55]. Evidence from adult-focused intervention studies demonstrates that structured community or clinic-based programs substantially increase vaccine uptake and series completion [56]. Embedding prompts within national cervical cancer registries could institutionalize this “same-day, same-site” model. Emotional reassurance also plays a crucial role; women who perceive vaccination as empowerment against recurrence show higher adherence [38,39].

International experience illustrates the dynamic role of coordination. The comprehensive nationwide program involving schools and opportunistic catch-up and screening of girls and women in Australia led to the near eradication of high-grade cervical precancer lesions within 15 years [21,40,41]. The decrease in HPV and CIN3+ in Scandinavia supports that population-level benefits can only be gained by comprehensive efforts at integration.

### 4.8. Ethical and Psychosocial Aspects

The principles of autonomy, beneficence, and justice form the backbone of immunization. Healthcare providers should thus respect the principle of autonomy by being transparent about the risks and benefits. It should be noted that HPV immunization prevents reinfection or reduces the incidence of recurrence but has not been used as a treatment for existing HPV disease [57].

Psychosocially, post-CIN women often experience anxiety, guilt, or fear regarding reproductive potential and sexual relationships. Qualitative studies reveal that supportive counseling mitigates distress and enhances perceived control [38,39]. Cultural sensitivity is crucial—while autonomy predominates in individualist societies, spousal or familial endorsement may be decisive in collectivist settings. Tailoring counseling to the local sociocultural context, using culturally adapted leaflets or survivor testimonials, improves acceptability. Reframing HPV vaccination as reproductive health promotion rather than an STI intervention helps destigmatize the topic and normalizes uptake [6,12,40].

Healthcare providers also face moral considerations at the community level. Vaccination supports the concept of justice by providing an equal opportunity to avoid HPV-related cancers. The main objective of immunization is to prevent cervical cancer, which still poses a high mortality rate in resource-poor nations.

### 4.9. Future Directions and Research Gaps

Although observational data provide strong evidence that CIN2+ lesions recur less often post-vaccination, randomized controlled trials have been scarce. In future trials, it would be helpful to see outcomes differentiated by HPV types, age groups, and types of vaccines used. In addition, psychosocial outcomes such as decreased anxiety levels, improved body images, and overall quality of life should also be separately assessed.

Future studies seeking to tease apart the differences in vaccine efficacy according to HPV genotype will be challenged by the following substantive issues. Firstly, the number of non-16/18 high-risk HPV types will be exceedingly low in the context of the vaccine pool, rendering it impractical to detect recurrence according to genotype. In particular, the effectiveness of existing vaccines will cloud the ability to identify the role of a specific genotype. Additionally, the vast majority of existing information regarding recurrence of CIN2+ will be insufficiently sized to represent the rarity of genotypes represented by HPV 31, 33, and/or 52. Lastly, genotype recurrence databases and uniform molecular testing will be needed to properly measure the effect of the vaccine according to HPV type.

International standardization of the vaccination policy post-treatment is a prerequisite. Currently, both ESGO and EFC guidelines advocate CIN2/3 treatment-related vaccinations for women, preferably within 12 months of excision, whereas the WHO and ACIP include women up to 45 years of age [27,51,52]. The creation of global registries addressing recurrence rates, fertility outcomes, and survival rates would thus provide comprehensive data for these recommendations.

Finally, the implementation of artificial intelligence-driven reminder services, digital immunization registries, and monitoring dashboard services may ensure smooth implementation with high coverage. The long-term objective of such screening efforts goes beyond the prevention of lesion recurrence. Rather, it aims at the eventual prevention of cervical cancer as a public health problem. This milestone can only be realized through equitable immunization strategies.

## 5. Conclusions

HPV vaccination in the context of treatment of CIN2+ lesions through excision provides important secondary prevention against the recurrence of high-grade lesions. However, there has been marked variance in its acceptance, driven by various factors at the individual level, against the backdrop of awareness of system-level access factors. This narrative review draws particular attention to the impact of physician support as the chief facilitating factor, rather than misinformation and cost as the chief hurdles.

The following topics should be explored in future research: (1) Risk of recurrence according to HPV type, (2) Comparative effectiveness of one versus two doses of vaccine in women who had received previous therapy, (3) Efficacy of electronic tools in correcting misinformation that hinders the decision-making process, and (4) Implementation strategies targeted at less resource-rich settings to eliminate inequities in the distribution of the vaccine. The above-mentioned research will be crucial to achieving the global target of cervical cancer elimination.

## Figures and Tables

**Table 1:** Geographic Patterns and Predictors of HPV Vaccine Acceptance.

Continent	Acceptance Range (%)	Key Barriers	Main Facilitators	Representative Studies
**Europe**	55–85	Low perceived need post-CIN, misinformation	Physician counseling, national programs	Elfström et al. [13]
**North America**	60–90	Cost, misinformation, mistrust	Insurance coverage, “teachable moment” effect	Smulian 2021 [14]; Brewer [15]
**Latin America**	40–80	Cultural stigma, fertility fears	Nurse-led education, free vaccination	Russ 2019 [16];
**Asia**	20–75	Governmental hesitancy (Japan), low awareness	Local vaccine availability, education	Hanley 2022 [17];
**Africa**	30–70	Access, cost, distrust	Integration with reproductive health programs	Makwe 2020 [18]; Msyamboza 2022 [19]
**Oceania**	80–95	Minimal (mainly logistics)	Universal funding, trust	Patel 2021 [20]; Brotherton 2020 [5]

## Supplementary Materials

The following supporting information can be downloaded at: https://www.mdpi.com/article/10.3390/jcm14248859/s1, Table S1: Full Search Strategy for the Narrative Review.
