# Supplementary material for: Perception and Acceptance of HPV Vaccination Among Women Treated for Cervical Intraepithelial Neoplasia: An Evidence-Based Narrative Review
Source: J Clin Med. 2025 Dec 15;14(24):8859. doi: 10.3390/jcm14248859 (PMC12734397; doi:10.3390/jcm14248859)
Supplement: Supplementary file 1 [file jcm-14-08859-s001.zip › jcm-4005931-supplementary.pdf]

Table S1. Full Search Strategy for the Narrative Review.

Database	Full Search String (as Executed)	Date Last Searched	Filters Applied	Notes
**PubMed**	(“human papillomavirus vaccine” [Mesh] OR “HPV vaccination” OR “HPV vaccine”) AND (“cervical intraepithelial neoplasia” OR CIN OR “cervical dysplasia” OR “cervical precancer”) AND (acceptance OR attitude OR perception OR awareness OR knowledge OR “vaccine hesitancy” OR determinant OR predictor)	10 March 2025	• Humans • English • 2010–2025	Complete PubMed string required by Reviewer 1
**Scopus**	TITLE-ABS-KEY (“HPV vaccination” OR “human papillomavirus vaccine”) AND TITLE-ABS-KEY (“cervical intraepithelial neoplasia” OR CIN OR “cervical dysplasia”) AND TITLE-ABS-KEY (acceptance OR attitude OR awareness OR perception OR determinant OR predictor)	10 March 2025	• English • Article/Review • 2010–2025	Exported references manually checked for duplicates
**Embase**	(‘human papillomavirus vaccine’/exp OR ‘HPV vaccination’ OR ‘quadrivalent vaccine’ OR ‘9-valent vaccine’) AND (‘cervical intraepithelial neoplasia’/exp OR CIN OR ‘cervical dysplasia’) AND (acceptance OR attitude OR perception OR ‘vaccine hesitancy’ OR awareness OR ‘decision-making’)	11 March 2025	• Human studies • English • 2010–2025	Emtree terms adapted from previous HPV systematic reviews
**Web of Science**	TS = (“HPV vaccination” OR “human papillomavirus vaccine”) AND TS = (“cervical intraepithelial neoplasia” OR CIN OR “cervical dysplasia”) AND TS = (acceptance OR perception OR attitude OR awareness OR determinant OR predictor)	11 March 2025	• Web of Science Core Collection • English • 2010–2025	Citation tracking performed manually
**Additional Sources**	—	—	• ESGO-EFC Guidelines • WHO HPV Technical Reports • ACIP/CDC Position Statements	Hand-searching of the reference lists of all included papers
